# Dose-Dependent Effect of Intravenous Administration of Human Umbilical Cord-Derived Mesenchymal Stem Cells in Neonatal Stroke Mice

**DOI:** 10.3389/fneur.2018.00133

**Published:** 2018-03-08

**Authors:** Emi Tanaka, Yuko Ogawa, Takeo Mukai, Yoshiaki Sato, Takashi Hamazaki, Tokiko Nagamura-Inoue, Mariko Harada-Shiba, Haruo Shintaku, Masahiro Tsuji

**Affiliations:** ^1^Department of Regenerative Medicine and Tissue Engineering, National Cerebral and Cardiovascular Center, Suita, Japan; ^2^Department of Pediatrics, Osaka City University Graduate School of Medicine, Osaka, Japan; ^3^Department of Cell Processing and Transfusion, Institute of Medical Science, The University of Tokyo, Tokyo, Japan; ^4^Division of Neonatology, Center for Maternal-Neonatal Care, Nagoya University Hospital, Nagoya, Japan

**Keywords:** mesenchymal stem cell, umbilical cord-derived mesenchymal stem cell, neonatal stroke, neonatal brain injury, intravenous administration

## Abstract

Neonatal brain injury induced by stroke causes significant disability, including cerebral palsy, and there is no effective therapy for stroke. Recently, mesenchymal stem cells (MSCs) have emerged as a promising tool for stem cell-based therapies. In this study, we examined the safety and efficacy of intravenously administered human umbilical cord-derived MSCs (UC-MSCs) in neonatal stroke mice. Pups underwent permanent middle cerebral artery occlusion at postnatal day 12 (P12), and low-dose (1 × 10^4^) or high-dose (1 × 10^5^) UC-MSCs were administered intravenously 48 h after the insult (P14). To evaluate the effect of the UC-MSC treatment, neurological behavior and cerebral blood flow were measured, and neuroanatomical analysis was performed at P28. To investigate the mechanisms of intravenously injected UC-MSCs, systemic blood flowmetry, *in vivo* imaging and human brain-derived neurotrophic factor (BDNF) measurements were performed. Functional disability was significantly improved in the high-dose UC-MSC group when compared with the vehicle group, but cerebral blood flow and cerebral hemispheric volume were not restored by UC-MSC therapy. The level of exogenous human BDNF was elevated only in the cerebrospinal fluid of one pup 24 h after UC-MSC injection, and *in vivo* imaging revealed that most UC-MSCs were trapped in the lungs and disappeared in a week without migration toward the brain or other organs. We found that systemic blood flow was stable over the 10 min after cell administration and that there were no differences in mortality among the groups. Immunohistopathological assessment showed that the percent area of Iba1-positive staining in the peri-infarct cortex was significantly reduced with the high-dose UC-MSC treatment compared with the vehicle treatment. These results suggest that intravenous administration of UC-MSCs is safe for a mouse model of neonatal stroke and improves dysfunction after middle cerebral artery occlusion by modulating the microglial reaction in the peri-infarct cortex.

## Introduction

Neonatal stroke is a common cause of acute neonatal encephalopathy and frequently results in neurological impairments such as cerebral palsy, cognitive disorders, and seizures (1, 2). The incidence of neonatal arterial ischemic stroke is 1 per 3,500 to 7,700 neonates, and the frequency of neonatal arterial ischemic stroke diagnosis has increased due to the advancement of imaging techniques (3, 4). Although neonates with stroke may present features of hypoxic-ischemic encephalopathy (HIE) (5, 6), therapeutic hypothermia has not currently been proven effective for neonatal stroke despite its efficacy in HIE. In addition, most neonatal stroke patients are recognized only retrospectively with emerging seizures or hemiparesis days after birth, hence the patients are likely to miss the opportunity of receiving acute neuroprotective treatments. There is currently no effective treatment for neonatal stroke. A novel therapeutic strategy to improve the outcome of neonatal stroke is needed.

Recently, many researchers have focused on cell therapies as novel treatments for neonatal brain injury. Our group has focused on the use of umbilical cord blood (UCB) for its advantage in feasibility to clinical applications (7), and previously we have shown the benefit of systemic administration of human UCB cells in animal models of neonatal encephalopathy (8, 9). Subsequently, we are conducting a clinical trial of autologous UCB transplantation for neonates with HIE (National Institutes of Health, ClinicalTrials.gov: NCT02256618). However, more than half of neonatal stroke patients do not present signs or symptoms at birth, hence physicians and parents may not recognize the need for preserving their own UCB. Even if the need is recognized, it is not always possible to collect their own UCB, especially when babies are unexpectedly born with severe asphyxia. An alternative cell source for neonatal brain injury is needed, and mesenchymal stem cells (MSCs) have emerged as a promising candidate. MSCs are isolated from umbilical cord, UCB, and placenta, as well as from bone marrow and adipose tissue, and they can be easily expanded in culture (10). Regardless of the cell source, each MSC population has a self-renewal capacity, a multi-lineage differentiation ability, and the potential for migration toward an inflamed or injured site (11, 12).

Umbilical cord-derived MSCs (UC-MSCs) have been proposed as a preferable cell source for regenerative medicine and immunotherapy, as they possess faster self-renewal, higher multipotency, and less immunogenicity than the features of born marrow-derived MSCs (13–16) and adipose-derived MSCs (17, 18). Considering these advantages and the demand for a cell source for neonates who do not have their own UCB, we are preparing to produce clinical grade off-the-shelf UC-MSCs for the next clinical application and for broad spectrum use in neonates with brain injuries. We have shown that intravenous administration of UC-MSCs ameliorates injuries via attenuating reactive gliosis and hypomyelination in a neonatal mouse model of intraventricular hemorrhage (IVH) (19). However, there is no study on UC-MSC in a neonatal stroke model. Regarding the injection route, most studies in models of neonatal brain injury have used local administration of MSCs, such as intraventricular and intranasal injection (20–23). There is a limited number of studies on intravenous MSC treatment for neonatal brain injury, one in sheep model (24) and four in rat models (25–28), and there is no study in mouse model except for our IVH report (19). With respect to studies on UC-MSCs for treatment of neonatal brain injury, most of them examined the effects of a focal injection (29–31). In clinical settings, intravenous administration is more desirable for unstable, sick neonates because it does not require an additional invasive procedure. Our previous study showed that cell distributions vary with injection routes, and more cells were trapped in the lungs when injected intravenously (32). Therefore, it is necessary to demonstrate the safety and efficacy of intravenous administration of UC-MSCs in neonatal animal models. In addition, studies of dosing regimens were limited mainly to UCB cells (33–36), evidence on intravenous MSC dosing is lacking. The main objective of this study was to investigate whether intravenous administration of UC-MSCs is safe and whether this treatment can attenuate brain damage and to determine the optimal dose for ameliorating the neurodevelopmental deficits after neonatal stroke.

## Materials and Methods

### Cell Preparation

Human umbilical cord tissues were obtained from women who underwent cesarean sections after informed consent was obtained. UC-MSCs were isolated as described by Mori et al. (37). Briefly, UC-MSCs were isolated by an improved explant method, and fragments were cultured with RM medium (kindly provided by ROHTO Pharmaceutical Co., Ltd., Osaka, Japan), which is a serum-free culture medium, at 37°C with 5% CO_2_. After confluence, adherent cells were trypsinized and replated (passage 1). The cells of fourth passage were cryopreserved in a cryoprotectant, STEM-CELLBANKER (ZENOAQ Resource Co., Ltd., Fukushima, Japan) which contains 10% dimethyl sulfoxide (DMSO). The expanded UC-MSCs were validated for their differentiation potential and cell surface molecules as previously described (38). Cells were rapidly thawed in a 37°C water bath just before use without washing. Of note, this cryoprotectant with DMSO has been proven safe for clinical use. When mostly thawed into liquid, the cells in a tube were kept on ice and used within 1–2 h. The viability of UC-MSCs was 91.75 ± 8.30%. The cryoprotectant was used as vehicle in the control group.

### Neonatal Stroke Model

All animal research studies were approved by the Experimental Animal Care and Use Committee of the National Cerebral and Cardiovascular Center and was done according to the NIH Guide for the Care and Use of Laboratory Animals.

**Figure 1:**
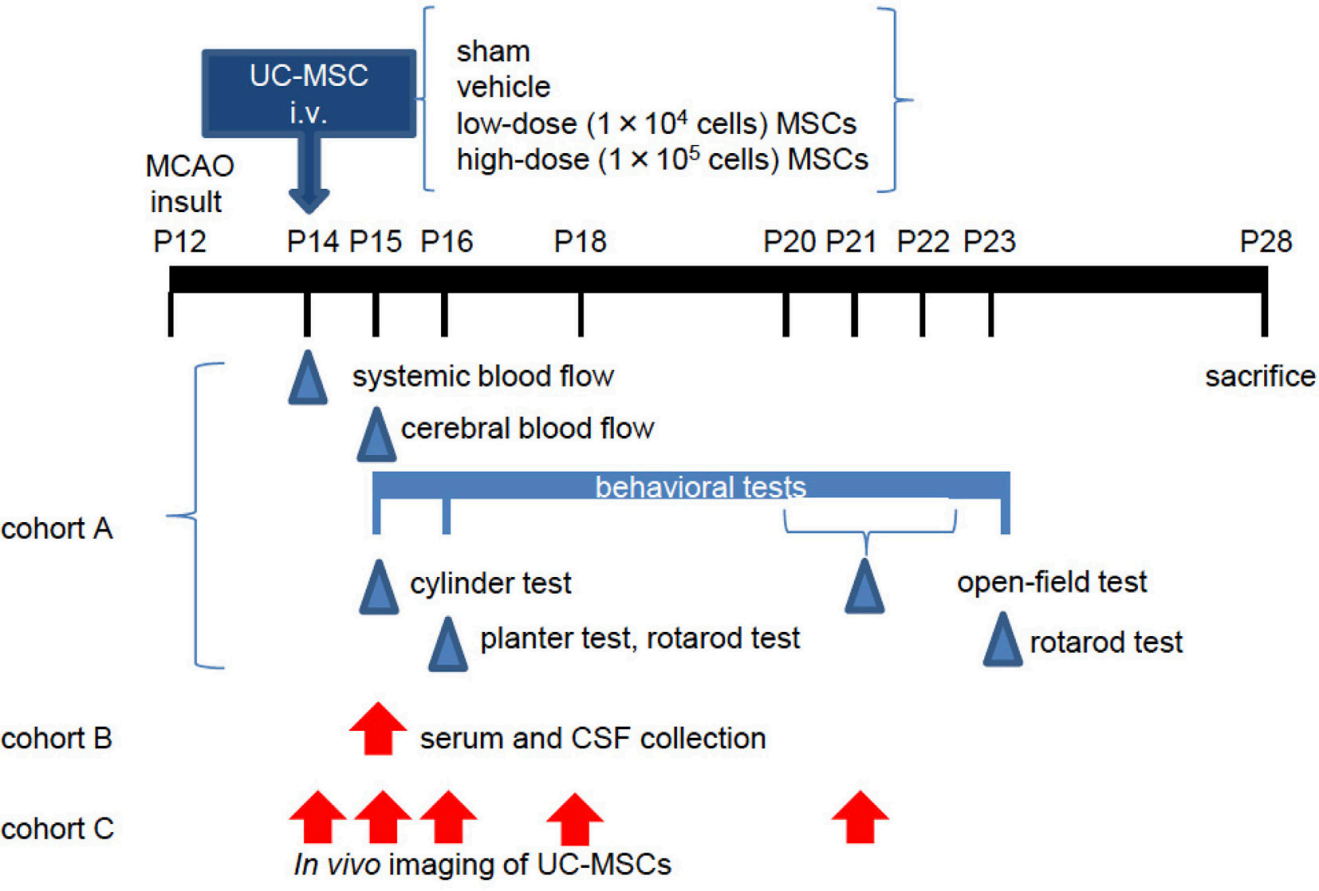
The experimental scheme. At postnatal day 12 (P12), mice underwent permanent middle cerebral artery occlusion (MCAO). Two days after the injury (P14), they were administered vehicle or umbilical cord-derived mesenchymal stem cells (UC-MSCs) intravenously (i.v.). (Cohort A) Systemic and cerebral blood flows were measured at P14 and P15, respectively. Neurological behavioral measurements were performed at P15–P23. After sacrifice, morphological and immunohistopathological assessments were conducted in this cohort. (Cohort B) Mouse serum and cerebrospinal fluid (CSF) were collected at P15 for evaluation of a trophic factor. (Cohort C) *In vivo* imaging was performed at different time points after UC-MSC administration: 3 h, 24 h, 48 h, 4 days, and 7 days after injection.

CB17 male and female mouse pups were used in the experiments (*n* = 90). Postnatal day 12 (P12) pups, which are thought to be equivalent to full-term human newborns at P0 (39, 40), were divided into a no-surgery control group (*n* = 6), a sham-surgery group (*n* = 12), and middle cerebral artery occlusion (MCAO) groups (*n* = 72). The pups were subjected to permanent MCAO as described previously (41). Under isoflurane anesthesia (4.0% for induction and 1.5–2.0% for maintenance), a hole was made in the left temporal bone. The left middle cerebral artery (MCA) was electrocauterized and disconnected just distal to its crossing of the olfactory tract. Pups in the sham group underwent open-skull surgery without MCA electrocoagulation. After the insult, mice were observed for any bleeding and was awakened in the 32°C infant-warmer and then returned to their dams. All mice tolerated the MCAO procedure, and there was no surgical mortality. The numbers of mouse pups used in each cohort are summarized in Table 1. The blood flow measurement, a battery of behavioral tests, and morphological evaluations were done in cohort A. A flow cytometry beads assay and *in vivo* imaging were done in cohorts B and C, respectively. All the other experiments were performed in cohort A (Figure 1).

**Table 1:** Numbers of mice used in the experiments.

Cohort	Control	Sham	Vehicle	Low-dose1 × 10^4^	High-dose1 × 10^5^
A		12	13	13	16
B	6		9		13
C			1		7

*Mice in cohort A were used for the blood flow measurement, behavioral, and histological evaluations; cohort B for blood and cerebrospinal fluid (CSF) sampling; and cohort C for *in vivo* imaging of injected cells*.

### Cell Administration

At 48 h after the MCAO procedure (at P14), mice were randomly divided into three groups: vehicle (*n* = 23), low-dose UC-MSCs (1 × 10^4^ cells, *n* = 13), and high-dose UC-MSCs (1 × 10^5^ cells, *n* = 36). From our previous study with a cell therapy (8) and from a clinical stand point, we thought that 48 h after the MCAO would be the optimal timing of cell administration for neonatal stroke; neonatal stroke is rarely diagnosed on the first day of life, rather diagnosed a few days after the birth in many cases. Pups in the UC-MSC groups were administered frozen-thawed UC-MSCs in 60 µl of STEM-CELLBANKER cryoprotectant. Pups in the vehicle group were administered cryoprotectant alone. After incising the skin in the left inguinal region, UC-MSCs were infused or vehicle was infused into the femoral vein using a 35-G needle under isoflurane anesthesia, slowly administered over 1 min as described previously (32). Under the microscope, we were able to directly observe the injection flow through the transparent vein when the needle insertion was optimal.

### Systemic and Cerebral Blood Flowmetry

Blood flow of the body surface and cerebral surface were measured by a laser speckle flowmetry imaging system (OmegazoneOZ-1, Omegaweve Inc., Tokyo, Japan). Flowmetry of the body surface as a representative of systemic blood flow was performed before and immediately after injection. First, under isoflurane anesthesia, pre-flow images were acquired every 5 s, and vehicle was administered or UC-MSCs were administered over 1 min followed by 30 s hemostasis with a swab. Second, post-flow images were sequentially taken every 5 s for 10 min. We set the region of interest in the chest, and the average blood flow per minute was compared with that from the pre-flow measurements (vehicle *n* = 7, low-dose *n* = 8, and high-dose *n* = 8).

**Figure 2:**
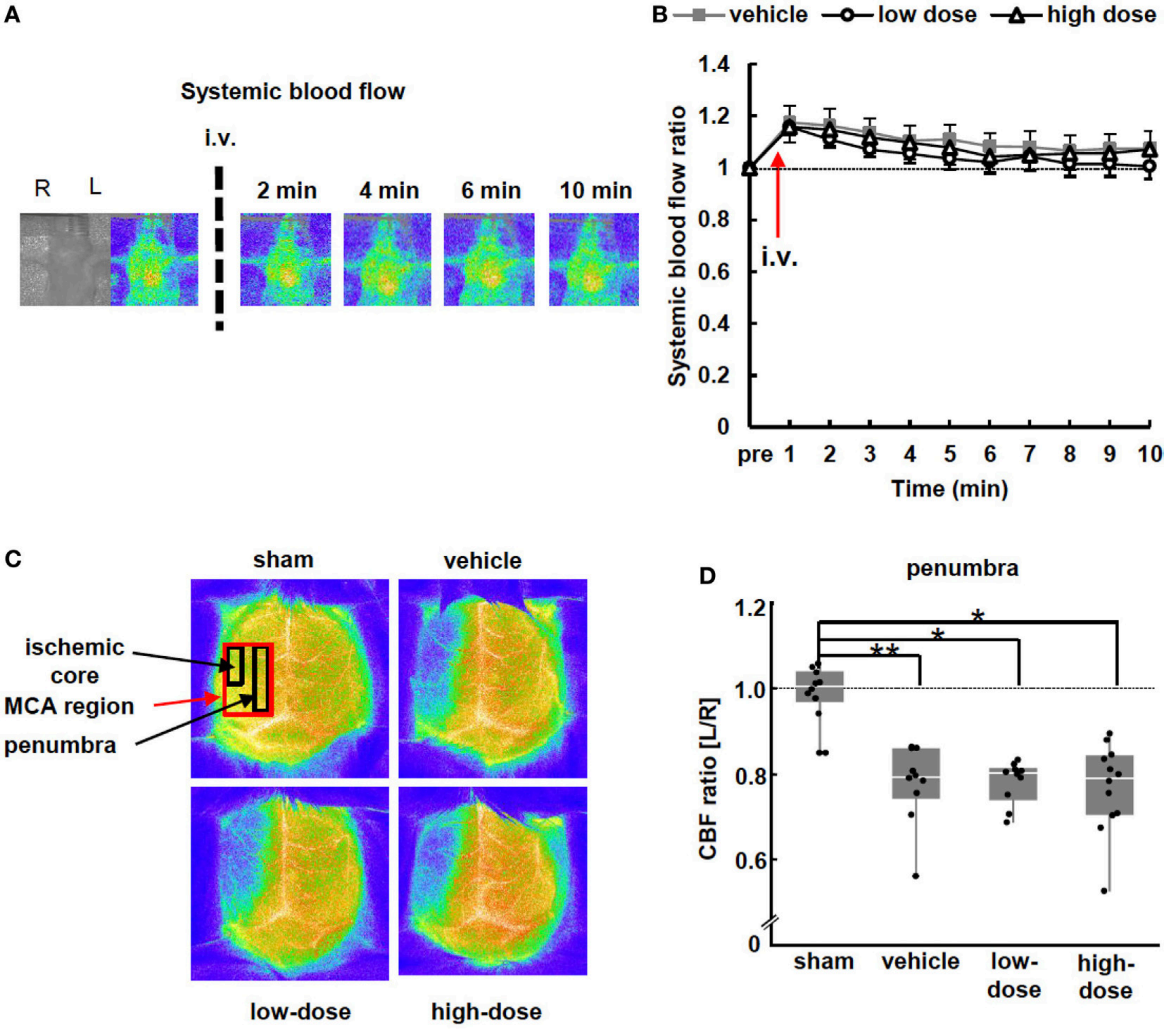
Blood flow measurements. **(A)** Representative images of systemic blood flow before and immediately after umbilical cord-derived MSC administration. **(B)** Systemic blood flow presented as a ratio to the pre-injection level. There were no significant differences between the vehicle and UC-MSC groups in systemic blood flow (vehicle *n* = 7, low-dose *n* = 8, and high-dose *n* = 8). **(C)** Representative images of cerebral blood flow (CBF) 24 h after administration. CBF decreased in the middle cerebral artery (MCA) region on the ipsilateral side of middle cerebral artery occlusion. **(D)** The CBF ratio of ipsilateral/contralateral hemispheres in the penumbra significantly decreased after MCAO. UC-MSC injection did not increase the CBF ratio. **p < 0.01 and *p < 0.05 (sham *n* = 10, vehicle *n* = 10, low-dose *n* = 10, and high-dose *n* = 12).

Cerebral blood flow (CBF) was measured 24 h after injection (at P15) as described previously (42). We evaluated the three ROIs such as ischemic core, penumbra and MCA region (Figure 2C). Data were presented as CBF ratio, ipsilateral/contralateral hemisphere (sham *n* = 10, vehicle *n* = 10, low-dose *n* = 10, high-dose *n* = 12).

### Behavioral Tests

To evaluate the effects on motor and sensory functions, the following four behavioral tests were performed: a cylinder test, a dynamic plantar test, a rotarod test, and an open-field test.

The cylinder test was performed on P15. Asymmetry of forelimb use was assessed during rearing in a transparent acrylic cylinder (43). We videotaped 20 rearing movements, counted wall touches by each forelimb separately, and analyzed forepaw use preference as follows: (nonimpaired side [left] − impaired side [right])/(nonimpaired + impaired sides) × 100 (sham *n* = 12, vehicle *n* = 18, low-dose *n* = 13, and high-dose *n* = 14).

The dynamic plantar test was performed on P16. We measured responses to von Frey filaments to assess sensory function as described previously (44, 45). Mice were acclimated in an elevated mesh floored cage and tested using the Dynamic Plantar Aesthesiometer (Ugo Basile, Varese, Italy). A filament was pushed up mechanically from beneath each paw, which increased in force gradually from 0 to 40 g over the course of 50 s until the mouse withdrew its paw. Each mouse received three trials per paw, the withdrawal time was noted, and the average was calculated as follows: (nonimpaired side − impaired side)/(nonimpaired + impaired sides) × 100 (sham *n* = 12, vehicle *n* = 13, low-dose *n* = 13, and high-dose *n* = 15).

For the rotarod test, sensorimotor skills were evaluated on P16 and P23. The rotarod accelerated from 4 to 40 rpm over 5 min (Ugo Basile, Varese, Italy) (8). The length of time that the mice remained on the rotarod was measured. Five measurements with longer than 3-min intervals were performed, and the average time was recorded.

For the open-field test, locomotor and exploratory behaviors were evaluated at 8–10 days after the insult (P20–P22) as described previously (8). Animals were allowed to act freely in a box (30 cm × 30 cm) for 30 min in the light and for 30 min in the dark (Taiyo Electric Co., Ltd., Osaka, Japan). Infrared beams were mounted at specific intervals on the X-, Y-, and Z-banks of the open-field area. The number of beam crossings by an animal was counted and scored as locomotion for the horizontal movement and as rearing for the vertical movement.

### Tissue Preparation and Morphology

A morphological evaluation of the brain was performed as previously described (46, 47). On P28, mice were sacrificed with pentobarbital i.p. and transcardially perfused with phosphate-buffered saline and then with 4% paraformaldehyde. Subsequently, the whole brain was removed and immersed overnight in the same fixative. Fixed brains were cut coronally into 1-mm slices and the hemispheric areas were measured using ImageJ software (National Institutes of Health, Bethesda, MD, USA). The hemispheric volume was estimated by integration of the hemispheric areas. The cerebral hemispheric volume ratio was calculated as follows: viable ipsilateral hemispheric volume/contralateral hemispheric volume.

### Immunohistochemistry

The processed brains were embedded in paraffin, and 5-µm coronal sections were prepared for every 1 mm. For immunohistochemical detection, anti-mouse GFAP (glial fibrillary acidic protein, a marker of astrocytes) antibody (1:100; Merck Millipore, Burlington, VT, USA), and anti-rabbit Iba1 (ionized calcium binding adaptor molecule, a marker of microglia 1) antibody (1:200; WAKO, Osaka, Japan,) were used. The secondary antibody was a goat-anti-mouse IgG polyclonal antibody (Nichirei Bioscience Inc., Tokyo, Japan). Slices were stained with 3,3′-diaminobenzidine (DAB) with hydrogen peroxide. The GFAP-positive astrocytes and Iba1-positive microglia were segmented by applying an appropriate threshold in gray value in order to distinguish from non-specific background staining. The percent areas (segmented area/total 400 μm × 400 μm area) were calculated using ImageJ software. The regions of interest (ROIs), i.e., square frame, were laid not to include tissue absent area or necrotic area by an examiner who was blinded to the experimental group of the mice. Four regions were determined from a section containing both the hippocampus and striatum; the peri-infarct cortex, hippocampus, subcortical white matter, and non-infarct cortex in an area of 400 μm × 400 μm. We calculated the percent area for two ROIs in one slice per region per animal (*n* = 5 per group).

### Flow Cytometry Beads Assay

In cohort B (Figure 1), the no-surgery control and MCAO mice were prepared. The MCAO mice were subjected to intravenous administration of vehicle or UC-MSCs (1 × 10^5^ cells; control *n* = 6, vehicle *n* = 6, and UC-MSCs *n* = 13). Mouse serum and cerebrospinal fluid (CSF) were collected 24 h after injection, and the concentration of human brain-derived neurotrophic factor (BDNF) secreted from human UC-MSCs was measured using a bead immunoassay as previously described (19). CSF was collected by puncture of cisterna magna with a 25-G needle and a capillary glass under microscope. Subsequently, blood was collected by cardiac puncture using a 1 ml syringe and a 26-G needle, and centrifuged to collect the serum. We quantified human BDNF with the HQ-Plex kit (Bay Bioscience Co., Ltd., Kobe, Japan). All samples were analyzed in triplicate according to the manufacturer’s instructions. Bead fluorescence readings were done by a BD^TM^ FACS Canto II flow cytometer (BD Biosciences, San Jose, CA, USA).

### *In Vivo* Imaging of UC-MSCs

In cohort C (Figure 1), eight MCAO mice received intravenous administration of vehicle (*n* = 1) or 1 × 10^5^ UC-MSCs (*n* = 7) 48 h after insult. UC-MSCs were traced using an IVIS Lumina II imaging system (Xenogen Corporation, Almeda, CA, USA) at 2 h, 24 h, 48 h, 4 days, and 7 days after the intravenous injection. Mice were given an intraperitoneal injection of d-luciferin (WAKO, Osaka, Japan) at a dose of 150 mg/kg 10 min before each imaging time point. Images were captured with an exposure time of 1 min under isoflurane anesthesia in a chamber, and images were analyzed using Living Image 3.0 software (Caliper Life Sciences, Waltham, MA, USA).

### Statistical Analysis

The mortality rate of the animals was analyzed using the Fisher’s exact test. Calculated values such as CBF ratios, the cylinder and plantar test results, cerebral hemispheric volumes, and percent areas of immunohistological staining were assessed using a Kruskal–Wallis test followed by Dunn’s test. Absolute values or temporal sequences that include systemic blood flow and the rotarod and open-field test results were assessed using two-way repeated measures ANOVA. The significance level was established at *p* < 0.05. The results are presented as the mean ± SEM in bar graphs or box plots (maximum, quartile point, medium, and minimum). All data were analyzed using JMP 12.2.0 software (SAS Institute, Cary, NC, USA).

## Results

### Mortality

In this study, 62 mice survived for two weeks after the insult, and seven died (Table 2). One mouse in the high-dose UC-MSC group died approximately 10 min after induction of anesthesia and a few minutes after cell administration. The rest died during a remote period approximately 7 days after the insult, indicating that the deaths may not be due directly to cell administration but rather due to weakness from the MCAO injury.

**Table 2:** Mortality rate.

Group	Time afterinjection	Sham	Vehicle	Low dose1 × 10^4^	High dose1 × 10^5^	
Dead/total (*n*)	24 h	0/12	0/23	0/13	1/36	n.s.
	2 weeks	1/12	1/14	1/13	4/23	n.s.

*Mortality rates at 24 h (postnatal day 15, P15) and 2 weeks (P28) after the vehicle or umbilical cord-derived mesenchymal stem cell (UC-MSC) injection were not different between the groups*.

### Blood Flowmetry

#### Systemic Blood Flow

Blood flow of the thoracic body surface was measured to assess systemic blood flow after cell administration. The blood flow in all groups was slightly increased and returned gradually to the pre-injection level after 10 min (Figures 2A,B). These changes in blood flow were almost concordant and were not different among the groups. This indicates that the systemic blood flow was not exacerbated by UC-MSC administration.

#### Cerebral Blood Flow

Twenty-four hours after cell injection (P15), the CBF was measured. CBF was decreased in the ipsilateral hemisphere of MCAO mice, and the decrease was significant in all assessed ROIs, including the ischemic core, penumbra, and MCA region (Figures 2C,D) compared with the CBF in the contralateral hemisphere. The UC-MSC injection did not increase the CBF. The two pups that showed the lowest and the second lowest CBF ratio, i.e., <0.6, died the week after the insult; one was in the vehicle group and the other was in the high-dose group.

### Behavioral Tests

**Figure 3:**
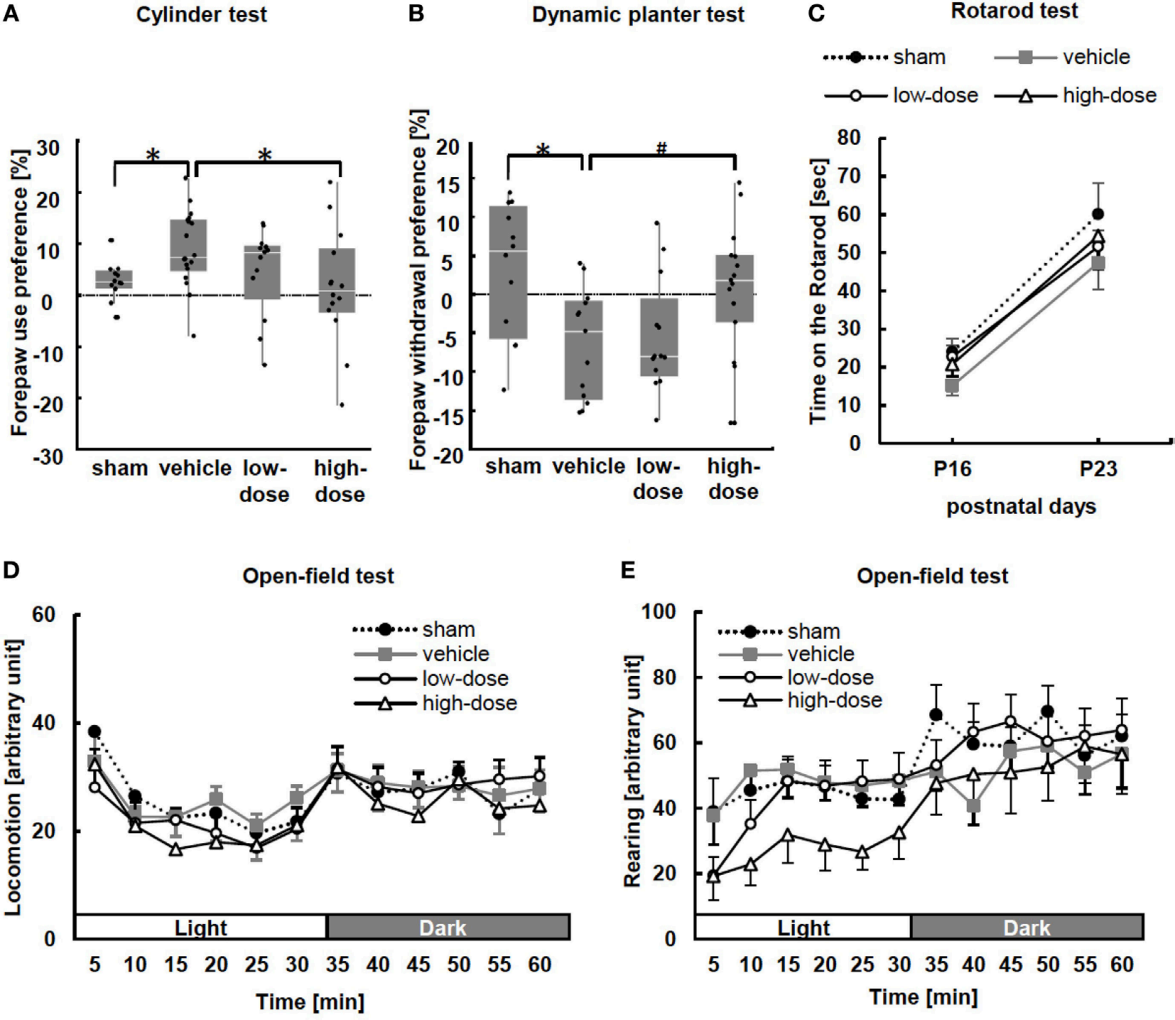
Behavioral tests: the cylinder test, dynamic plantar test, rotarod test, and open-field test. **(A)** In the cylinder test performed at P15, significant asymmetrical forepaw use was observed in the vehicle group. After administration of high-dose umbilical cord-derived MSCs, forepaw preference significantly improved. Wall touches by the left of right forepaw were measured separately, and paw preference with wall touches was calculated as (nonimpaired side [left] − impaired side [right])/(nonimpaired + impaired sides) × 100. *p < 0.05 (sham *n* = 12, vehicle *n* = 18, low-dose *n* = 13, and high-dose *n* = 14). **(B)** The dynamic plantar test was performed at P15 to assess the extent of sensory deficit. The forepaw of vehicle mice showed significant asymmetrical dullness compared with that of the sham mice. The high-dose UC-MSC mice showed a trend toward improvement. Average withdrawal time was calculated as (nonimpaired − impaired)/(nonimpaired + impaired) × 100. *p < 0.05 and ^#^*p* = 0.092 (sham *n* = 12, vehicle *n* = 13, low-dose *n* = 15, and high-dose *n* = 15). **(C)** In the rotarod test at P16 and P23, there were no significant differences among the groups (P16: sham *n* = 12, vehicle *n* = 13, low-dose *n* = 13, and high-dose *n* = 15; and P23: sham *n* = 10, vehicle *n* = 12, low-dose *n* = 13, and high-dose *n* = 11). **(D,E)** The open-field test was performed from P20 to P22. There were no significant intergroup differences with respect to either locomotion, i.e., spontaneous horizontal movement, or rearing, i.e., spontaneous vertical movement (*n* = 11–13 in each group).

We observed forelimb asymmetry to evaluate motor function by the cylinder test at P15. In this test, sham mice did not show paw preference (Figure 3A). MCAO caused significant asymmetry to use the left unimpaired forepaw in the vehicle group. After administration of high-dose UC-MSCs, performance in the cylinder test was significantly improved in comparison to the performance of the vehicle mice.

At P16, the dynamic plantar test was performed. Delayed withdrawal time from the filament was measured in forepaws and hind paws to assess the extent of sensory deficit. The changes were more obvious in the forepaw performance (Figure 3B). The vehicle mice showed significant asymmetrical dullness in the forepaw compared with that in the sham mice. Although the mice treated with UC-MSCs did not show significant improvement in performance compared with that in the vehicle mice, the high-dose UC-MSC mice showed a trend toward improvement (*p* = 0.092) in forepaw performance. There was no significant difference in hind paw sensory preference between the sham and vehicle groups (data not shown).

We performed the rotarod test at two time points: P16 and P23. The average number of times fallen from the rotarod cylinder shows sensorimotor capacity. There were no significant differences among the groups (Figure 3C).

Additionally, we evaluated spontaneous activity in an open-field test during P20–22. There were no differences among the groups (Figures 3D,E).

### Morphology

**Figure 4:**
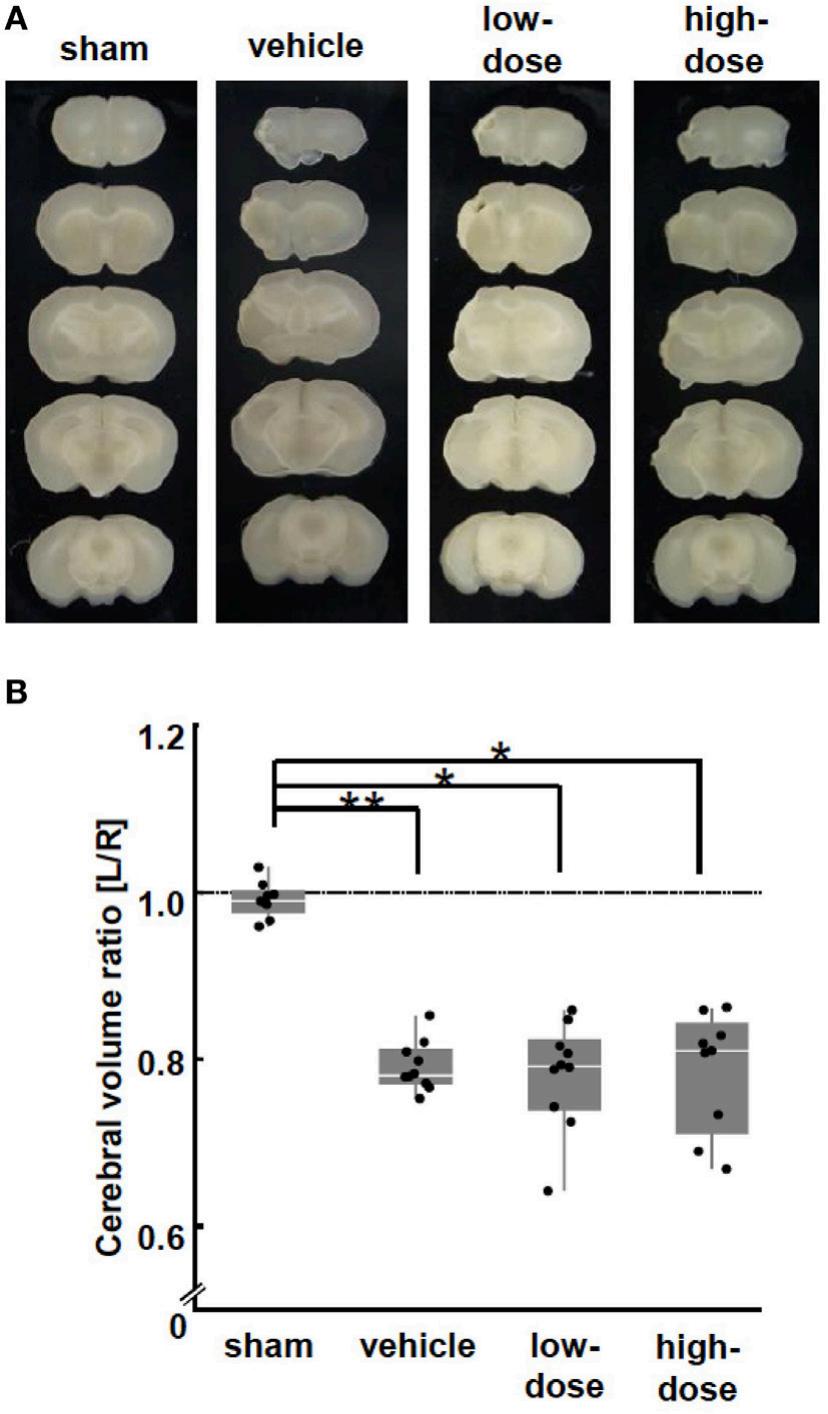
Morphological brain injury. **(A)** Representative images of brain coronal sections at P28 (2 weeks after the cells or vehicle administration). **(B)** At P28, middle cerebral artery occlusion caused a significant volume loss in the ipsilateral hemisphere in the vehicle group. There was no difference in the volume loss between the UC-MSC group and the vehicle group. **p < 0.01.

Cerebral hemispheric volume was estimated in fixed brain tissue. The volumes were consistent across all animals in the sham group (Figures 4A,B). MCAO caused a significant volume loss in the vehicle group. There was no improvement in the volume by UC-MSC administration compared with the volume in the vehicle group.

### Immunohistopathology

**Figure 5:**
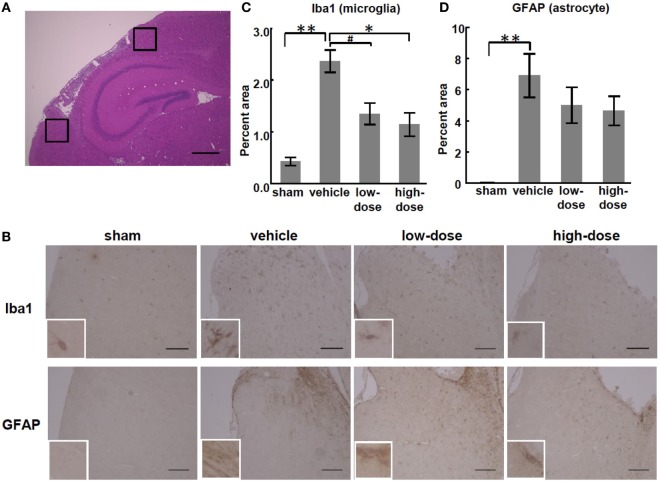
Effect of UC-MSC administration on the expression of glial cell markers in the peri-infarct cortex. **(A)** A representative photomicrograph of a brain coronal section stained with hematoxylin-eosin at P28. The black squares illustrate the regions that were quantified as the peri-infarct cortex. Bar, 500 µm (×100). **(B)** Representative photomicrographs of the area of the peri-infarct cortex at P28. Bar, 100 µm (×200). Inset shows higher magnification (×600). **(C)** The percent area of cells that stained positive for Iba1 was significantly higher in the vehicle group than in the sham group. There was a significant decrease in the high-dose UC-MSC group and a trend toward a decrease in the low-dose UC-MSC group. **(D)** Similarly, the percent area of cells that stained positive for glial fibrillary acidic protein (GFAP) was significantly higher in the vehicle group than in the sham group, but it was not significantly decreased in the UC-MSC groups than in the vehicle group. **p < 0.01, *p < 0.05, and ^#^*p* = 0.083.

We observed inflammation in sections of the injured brains at P28. MCAO significantly increased the percent areas of GFAP- and Iba1-positive cells in the peri-infarct cortex and subcortical white matter in the vehicle group compared with those in the sham group. There was no significant increase in the areas of the hippocampus or non-infarct cortex of the vehicle group. In the peri-infarct cortex, high-dose UC-MSCs significantly reduced the Iba1-positive percent area, and low-dose UC-MSCs also showed the same trend toward reduction (*p* = 0.083). Therefore, this result shows that high-dose UC-MSCs decreased the microglial accumulation in the peri-infarct cortex (Figure 5).

### Detection of Human BDNF *In Vivo*

**Figure 6:**
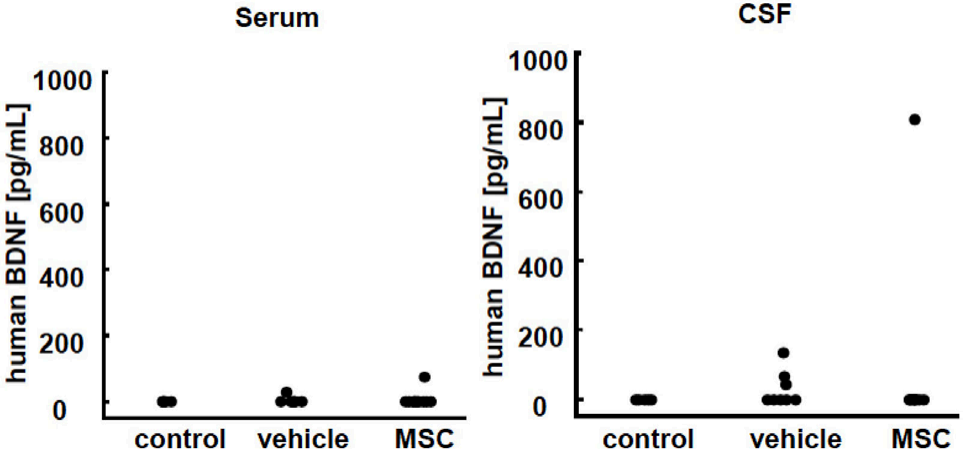
Human brain-derived neurotrophic factor (BDNF) detection in serum and cerebrospinal fluid (CSF) 24 h after injection. No BDNF was detected in the control group. Only one of the 13 mice treated with umbilical cord-derived MSCs showed elevated human BDNF levels in CSF. Some mice in the vehicle group showed a minimal equivocal level of BDNF (control *n* = 6, vehicle *n* = 6, and UC-MSC *n* = 13).

We assessed the presence of the neurotrophic factor BDNF from human UC-MSCs. Both serum and CSF were assessed 24 h after injection (P15). There was no detection of BDNF in the control group (Figure 6). Only one of the 13 mice treated with UC-MSCs showed an elevated level of human BDNF in CSF. A few mice had minimal equivocal levels of BDNF, which were inconclusive as to whether the observed increases were meaningful.

### *In Vivo* Imaging

**Figure 7:**
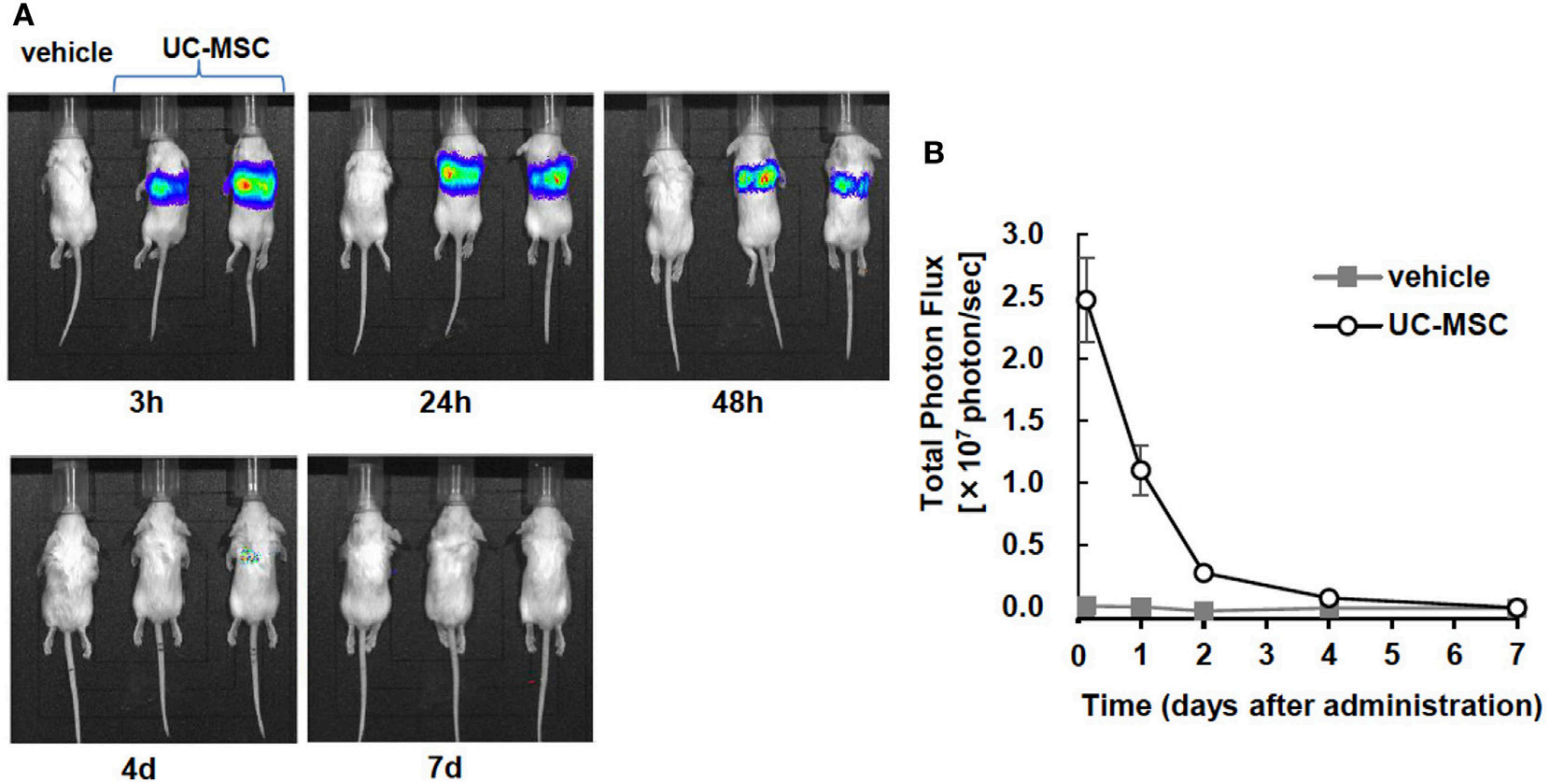
*In vivo* imaging of intravenously injected umbilical cord-derived MSCs (UC-MSCs). **(A)** Representative images of *in vivo* imaging taken at 3 h, 24 h, and 48 h and 4 days and 7 days after the intravenous injection of UC-MSCs. **(B)** Quantification of the total photon flux in the vehicle- and UC-MSC-treated groups. UC-MSCs were trapped in the lungs, and the intensity of photon flux decreased over time. We did not detect the signal in brain tissue (vehicle *n* = 1 and UC-MSC *n* = 7).

An *in vivo* imaging system was used to quantify the photon flux in the vehicle- and UC-MSC-treated groups at 3 h, 24 h, 48 h, 4 days, and 7 days after injection. The images revealed that intravenously injected UC-MSCs were rapidly trapped in the lungs. The intensity of photon flux was highest in the first image acquisition of the UC-MSC mice and then decreased over time, disappearing by 7 days. We did not detect the signals in other organs such as the brain or spleen (Figure 7).

## Discussion

In this study, we demonstrated that intravenous administration of human UC-MSCs following MCAO in neonatal mice was safe and attenuated damages in neurodevelopmental behaviors and glial cell reaction after neonatal stroke. We found that systemic blood flow was stable during 10 min after cell administration despite most UC-MSCs being trapped in the lungs and that there were no differences in mortality among the groups. Although the CBF and cerebral hemispheric volume were not restored after UC-MSC administration, neurological performance was significantly improved.

This is the first report from the following two standpoints: (1) UC-MSCs were used in a neonatal stroke model, and (2) two cell doses were compared in a scheme of intravenous MSC injection for neonatal brain injury. To our knowledge, there is no study on the use of UC-MSCs for neonatal stroke. There are a few studies in the literature that examined the effects of UC-MSCs for neonatal brain injury and showed functional recovery (19, 27–30). As there is no proven effective therapy for neonatal stroke, we investigated whether UC-MSCs improve neurological outcomes of neonatal stroke.

No report has demonstrated an optimal dose of intravenous MSCs injection in a neonatal brain injury model. Our current study aimed to determine an optimal MSCs dose for neonatal brain injuries that can be translated into clinical settings. To this end, two different doses of UC-MSC were compared for their effectiveness and safety in the behavioral tests and histological assessments. The higher dose was chosen based on the results of our previous study where intravenous administration of 1 × 10^5^ human UCB CD34^+^ cells was found beneficial in the same mouse model of neonatal stroke (8). The lower dose of 1 × 10^4^ cells was chosen to see whether these beneficial effects could be obtained even after lowering dosage. In light of clinical translation, lower dose is considered preferable because of limited availability of these cells and cost. Although there were no differences in the CBF or morphology, mice treated with the high-dose of UC-MSCs (1 × 10^5^ cells) showed a significant improvement in neurological function and glial responses after MCAO, and mice treated with the low-dose of UC-MSCs (1 × 10^4^ cells) showed a trend toward improvement. Studies of MSC dosing are limited only to focal injection (48–50); Donega et al. showed that 0.5 × 10^6^ bone marrow-derived MSCs was the minimal effective dose in HIE model mice when administered intranasally. With regard to mononuclear cells (33–36), there are some reports of intravenously injected cells which also show dose effects. Taken together, a relatively high dose of UC-MSCs may be needed to exert therapeutic effects in neonatal stroke mice.

Another strength of this study is the route of intravenous administration to neonatal mice. This study showed that intravenously injected UC-MSCs did not increase mortality or decrease systemic blood flow in these small mice; although, *in vivo* imaging demonstrated that the administered cells were mainly trapped in the lungs. Intravenous administration of UC-MSCs is studied in many clinical trials in adults without major adverse events (51). However, considering the size and adhesive characteristics of MSCs, there is a concern about whether the intravenous injection is safe for clinically unstable newborns. Six reports were published that studied intravenous injection of MSCs in models of neonatal brain injury (19, 24–28). Our previous report is the only study performed in mice (19). Similar to the immunohistological evaluation in our previous study, *in vivo* imaging in the present study demonstrated that intravenously injected US-MSCs were mainly trapped in the lungs (32). We were concerned about the possibility of a pulmonary embolism caused by the administered cells, so we measured systemic blood flow before and after the intravenous injection to rule out that possibility. Although a study of large animals demonstrated that the oxygen saturation after intravenous transplantation of MSCs was not significantly changed (52), there is no such study in neonatal mouse model. The stability in systemic blood flow suggests that the intravenous injection of UC-MSCs does not cause significant blood vessel embolism and is safe for newborn humans as well.

We studied how intravenously injected UC-MSCs distribute and act in the injured brain. Contrary to expectations, only one of the 13 mice treated with human UC-MSCs exhibited an increase in human BDNF in serum and CSF, and *in vivo* imaging showed that no animal exhibited migration of UC-MSCs toward the brain. We previously showed that intravenous administration of human UC-MSCs increased the levels of human BDNF and nerve growth factor in serum and CSF in about half of the mouse pups that were subjected to IVH at P5 (19). The previous results demonstrated the time-dependent migration of UC-MSCs toward the brain at 3 days after the injection, whereas this study did not detect the cell migration toward the brain. This contradiction may be due to differences in the pathophysiology (hemorrhage-induced vs. ischemia-induced injury) and the ages of animals used. Extracellular hemoglobin and their metabolites from IVH cause inflammation and contribute to mRNA up-regulation of pro-inflammatory cytokines after 72 h (53). These cytokines increase blood-brain barrier (BBB) permeability, leading to a consequent increase in endothelial adhesion molecules and modifications of the molecular composition/functional state of tight junctions. In contrast, a report showed that MCAO-induced BBB dysfunction was rather moderate and stable than that by LPS- or cold-induced injury (54); although, the report did not directly compare the IVH and MCAO methods. We measured human BDNF excreted from exogenous UC-MSCs but not endogenous murine BDNF. Ahn et al. showed a protective effect of human UCB-derived MSCs and an increase in endogenous BDNF expression 2 and 5 days after an intraventricular injection to IVH model rats (55). Taken together, the variety of inflammatory responses among different models critically influences the fate and actions of MSCs, and therapeutic effect may be induced mainly by the endogenous response in the recipient and not by direct action of the donor cells.

As there was no evidence of migration toward the injured brain tissue or restoration in CBF, we do not consider that cell replacement and improved cerebral perfusion contributed to the neurological improvement in this study. Similar to our current results, studies in the literature often show behavioral improvement without neuroanatomical improvement. One of the mechanisms by which UC-MSCs exert neuroprotective/neurorestorative effects was via a significant reduction in microgliosis in the peri-infarct cortex by high-dose UC-MSC injection, which modulates neuro-inflammation. Although cell replacement is considered a pivotal component in stem cell therapies including MSC therapies, accumulating studies have focused more on immunomodulation of the inflammatory microenvironment (56). Zhu et al. injected UC-MSCs intraperitoneally to a neonatal model of periventricular white matter damage and showed a decrease in reactive astrocytes and activated microglia in white matter (29). In post-ischemic tissue, M1 microglia are dominant and contribute to the inflammatory cascade and propagate cell death beyond the initial ischemic region (57, 58). With regard to macrophages, MSCs release factors that switch them from pro-inflammatory type 1 to an anti-inflammatory type 2 phenotype (59, 60) and produce TGF-β, which promotes the induction of Treg cells, ultimately leading to immune tolerance (61). Donega et al. demonstrated that transplanted MSCs modulate microglia to the immunosuppressive M2 type in neonatal ischemic brain injury (49). Our results also indicate that a reduction in microgliosis by intravenously injected UC-MSCs modulates the inflammatory cascade, which may contribute to the therapeutic efficacy rather than cell replacement. Further studies are needed to clarify mechanisms of stem cells actions, which may include electrophysiological and microstructural studies.

In conclusion, intravenous administration of UC-MSCs was safely performed in the small animal model of neonatal stroke mice, and the high dose (1 × 10^5^) of UC-MSCs improved functional outcomes. We consider that one of the underlying mechanisms of the therapeutic effect was microglial immunomodulation.

## Ethics Statement

All animal research was approved by the Experimental Animal Care and Use Committee of the National Cerebral and Cardiovascular Center and was done according to the NIH Guide for the Care and Use of Laboratory Animals.

## Author Contributions

ET performed the experiments (behavioral tests, etc.), analyzed data, prepared the figures, and wrote the manuscript. YO and TM performed the experiments (behavioral tests, etc.) and contributed to the analysis and interpretation of data. MT designed the study, performed the experiments (animal model, etc.), and critically reviewed the manuscript. YS, TH, TN, MS, and HS supervised the project and revised the manuscript critically for important intellectual content. All authors approved the manuscript.

## Conflict of Interest Statement

The authors declare that the research was conducted in the absence of any commercial or financial relationships that could be construed as potential conflicts of interest.
